# Haplotype-based quantitative trait mapping using a clustering algorithm

**DOI:** 10.1186/1471-2105-7-258

**Published:** 2006-05-18

**Authors:** Jing Li, Yingyao Zhou, Robert C Elston

**Affiliations:** 1 Electrical Engineering and Computer Science Department, Case Western Reserve University, Cleveland, OH 44106, USA; 2 Genomics Institute of the Novartis Research Foundation, San Diego, CA 92121, USA; 3 Department of Epidemiology and Biostatistics, Case Western Reserve University, Cleveland, OH 44106, USA

## Abstract

**Background:**

With the availability of large-scale, high-density single-nucleotide polymorphism (SNP) markers, substantial effort has been made in identifying disease-causing genes using linkage disequilibrium (LD) mapping by haplotype analysis of unrelated individuals. In addition to complex diseases, many continuously distributed quantitative traits are of primary clinical and health significance. However the development of association mapping methods using unrelated individuals for quantitative traits has received relatively less attention.

**Results:**

We recently developed an association mapping method for complex diseases by mining the sharing of haplotype segments (*i.e*., phased genotype pairs) in affected individuals that are rarely present in normal individuals. In this paper, we extend our previous work to address the problem of quantitative trait mapping from unrelated individuals. The method is non-parametric in nature, and statistical significance can be obtained by a permutation test. It can also be incorporated into the one-way ANCOVA (analysis of covariance) framework so that other factors and covariates can be easily incorporated. The effectiveness of the approach is demonstrated by extensive experimental studies using both simulated and real data sets. The results show that our haplotype-based approach is more robust than two statistical methods based on single markers: a single SNP association test (SSA) and the Mann-Whitney U-test (MWU). The algorithm has been incorporated into our existing software package called HapMiner, which is available from our website at .

**Conclusion:**

For QTL (*quantitative trait loci*) fine mapping, to identify QTNs (*quantitative trait nucleotides*) with realistic effects (the contribution of each QTN less than 10% of total variance of the trait), large samples sizes (≥ 500) are needed for all the methods. The overall performance of HapMiner is better than that of the other two methods. Its effectiveness further depends on other factors such as recombination rates and the density of typed SNPs. Haplotype-based methods might provide higher power than methods based on a single SNP when using tag SNPs selected from a small number of samples or some other sources (such as HapMap data). Rank-based statistics usually have much lower power, as shown in our study.

## Background

With the completion of the human genome project, substantial effort has been made to identify all common genetic variations, such as single nucleotide polymorphisms (SNPs), from different populations in order to have a detailed understanding of heritable variation in the human genome. While millions of SNPs have been identified, a grand challenge in the post genomic era is to develop robust strategies for identifying genetic contributions to complex traits that are important to human health, using SNPs as genetic markers for whole genome-scale wide analyses or fine-scale mapping. Complex traits, including most common diseases and many continuously distributed quantitative traits, are usually determined by multiple genetic and environmental factors, and potentially gene-gene interactions and gene-environment interactions. The challenge of identifying and fine-mapping genes underlying complex traits arises for many reasons, including the complexity of the genetic architecture of a trait, the small genetic effects which require a very large sample size, the difficulty of defining appropriate phenotypes, and the lack of effective approaches, among others. Nevertheless, vigorous progress has been made in advancing the understanding of the haplotype structure of human populations and in developing novel methodologies for genomic association mapping of disease genes using haplotype information. For case-control designs, the key assumption underlying haplotype mapping is the nonrandom association of alleles in disease haplotypes around the disease genes. The haplotypes from cases are expected to be more similar than haplotypes from controls in the regions near the disease genes. Various statistical methods (*e.g*., [10,19,21,22,29]) have been proposed to take advantage of information about shared haplotype segments instead of individual markers because the former type of information may provide higher power and greater accuracy. Strategies inspired by data mining techniques (*e.g*., [18,28]) have also been proposed as alternatives to model-based statistical methods.

In addition to binary traits, many continuously distributed quantitative traits are of primary clinical and health significance. Examples of such quantitative traits are blood pressure, cholesterol level, and bone mineral density. In many cases, the disease status of an individual is actually defined based on some threshold value of a particular quantitative trait. The traditional linkage methods for QTL mapping are primarily based on family data (see [15] for a more detailed treatment of QTL mapping). The extension of TDT-type (*i.e*., *Transmission Disequilibrium Test*) methods to QTL association mapping [3,8,9] also requires family information. The development of association mapping methods using unrelated individuals for quantitative traits has received relatively less attention [12]. But quantitative values can actually provide much more detailed information than the disease status alone and are collected routinely in most studies. Owing to the increasing interest in genomic association studies of complex diseases, there are also increasingly available quantitative data from unrelated individuals. Therefore, there is a great need for the development of novel algorithms that could directly map quantitative traits using population samples. In a recent paper, we described a novel algorithmic approach for haplotype mapping of disease genes that utilizes a clustering algorithm([18]). We reason that *disease susceptibility *(DS) allele embedded haplotypes, especially mutants of recent origin, tend to be close to each other due to linkage disequilibrium, while other haplotypes can be regarded as random noise sampled from the haplotype space. The algorithm considers haplotype segments as data points in a high dimensional space. Clusters are then identified using a density-based clustering algorithm [11]. Pearson *χ*^2 ^statistic or a *Z*-score based on the numbers of cases and controls in a cluster can be used as an indicator of the degree of association between the cluster and the disease under study. We introduced the concept of "density-based" clusters that was shown to be critical to its effectiveness, owing to the nature of the noisy data. In this study, we extend our previous method to QTL association mapping based on haplotype information from unrelated individuals. Clusters will be identified first using the density-based clustering algorithm. The degree of association of a cluster and the quantitative trait is measured by a *Q*-score, which is based on the *t*-statistic for testing the mean difference between two groups. The method can also be incorporated into the one-way ANCOVA framework so that other factors and covariates can be easily included in the analysis. The method is non-parametric in nature, because the significance of the predictions will be validated using permutation tests. Like its counterpart for disease mapping, the effectiveness of the approach depends on the similarity measure of haplotype fragments used in the clustering algorithm. We use the haplotype similarity measure proposed in [18], which both captures the sharing of haplotype segments due to historical recombination events and incorporates recent mutations and/or genotype errors.

To systematically evaluate the proposed algorithm, we perform extensive experimental studies using simulated and real data sets. We investigate the power of the proposed algorithm, defined as the proportion of times a significant association is detected from *n *(*n *= 200 in our experiments) independent replicates, and compare our method to two other statistical approaches based on single marker information. One is the most commonly used association test based on allele states of a single SNP (SSA), which is actually a one-way ANOVA analysis for quantitative traits. The other is a nonparametric test based on the Mann-Whitney U-test (MWU) [24]. It has been shown [4] that this rank-based statistic has better performance than SSA in detecting QTL associations. In addition to power, we are also interested in the localization accuracy of each method, measured by the distance from the SNP with the greatest score to the true QTN. To generate simulated data sets, we have adopted the coalescent model of evolution. The coalescent model provides an efficient way of investigating the effects of population parameters, such as recombination rates, on the power of an association method and has been commonly used in studying the properties of association approaches [4,20,31]. For our purpose, a candidate gene region with different numbers of SNPs will be generated using realistic recombination rates and mutation rates. A causative DNA polymorphism will be selected randomly from all the SNPs. The effect of the QTN contributes a varying proportion of the total variation of a quantitative trait. The simulation based on the coalescent theory is a great tool for investigating the influence of population parameters on the power of new approaches in a controlled manner, but it might not capture the true characteristics of the LD in human populations. We therefore further test the proposed algorithm on empirical (rather than simulated) human data. We take the phased genotype data of all parents from [6], and compare our algorithm with the other two methods on the complete data set as well as on selected tag SNPs. Results on both simulated and real data sets will be presented in the next section, followed by some discussion on possible future directions. The details of the algorithm are presented in the Method section.

## Results

### Model for generating simulated data

The coalescent model has been widely used in assessing the power of association tests [4,20,31]. It assumes a random mating population with selectively neutral mutations and an infinite-sites model. It is believed that the model could generate samples that are reasonable approximations of human variation with respect to the density, number and frequency spectrum of SNPs, and the pattern of linkage disequilibrium between them [4,20], although it involves some simplifications in mimicking human populations. In our simulation, the MS program from [14] was utilized to generate a large number of independent replicates of genealogies and SNPs under a wide range of population parameters. To examine the power of the three methods, four parameters that have potential effects on the power were investigated, namely, the sample size, the QTN effect, the recombination rate, and the density or number of SNPs. The size of the region for fine mapping was fixed to be 50 kb, with an effective population size (*N*_*e*_) of 10^6^. The total number of SNPs was set to be 50 or 100, corresponding to the density of 1 SNP per 1000 or 500 base pairs, respectively. Only SNPs with minor allele frequency larger than 5% were included in the calculation, so the actual number of SNPs (*n*) in each replicate varied. Haploid data were used in the study to avoid the time needed for haplotype inference. Similar results can be expected to hold for diploid data under the additive model. The sample sizes (*m*) considered were 250, 500, 750. Recombination rate has a strong influence on the power of LD-based tests. We used realistic values of recombination rates based on the data of human populations. The evolutionary recombination rates (*c*) between adjacent sites considered were 0.5E-9, 1E-9, 2E-9 per individual per generation, corresponding to a rate (*u*) of 100, 200, 400 at the population level in the region (*u *= 4*N*_*e*_*c *× size of the region), respectively. After each replicate was generated, a SNP was randomly chosen as a QTN, and was deleted from the data before further analysis. The effect of the QTN (*π*) was defined as the proportion of phenotypic variation attributable to the QTN. In most complex traits in human, a realistic estimate of *π *for a single QTN is usually less than 0.1. The power of any statistical approaches to detect such a QTN could be quite low [20]. In our study, the contribution of the QTN to the total variation of the quantitative trait was set to 0.1, 0.05, and 0 (for type I error). We have taken conservative values of *π *because they are more likely to represent reality, and the power of detecting associations with larger *π *would be higher. The model used to generate the phenotypic distribution was similar to that used in [4,20]. Details will be illustrated in the Method section. For each parameter combination, 200 independent replicates were generated. For each replicate, a permutation test with 1000 shuffles was performed to obtain the experimentwise significate level [5].

### HapMiner parameters

There are five parameters that need to be specified in HapMiner. It has been shown that HapMiner is quite robust and has consistent performance across a wide range of parameter values in disease gene mapping [18]. In this study, the two weight functions were assigned to be the strength of pairwise linkage disequilibrium measured by *D'*, for the reasons to be discussed in the Method section. The haplotype segment length was seven for both the simulated data and the real data with complete SNPs. The length was three for the real data with tag SNPs. The other two parameters for the clustering algorithm took their default values.

### Type I error

To assess the power of different approaches of detecting significant associations between SNPs and traits, it is important to have a proper control of false positive discoveries due to chance (*i.e*., type I errors). In this study, we set the error rate to be 0.05. The false positive rate of each method was estimated as the proportion of significant associations reported in 200 independent replicates for each parameter combination while keeping the contribution of the QTN to be 0. All three methods have correct type I error rates. The average false positive rates (and their standard errors) over all parameter combinations tested (*i.e*., sample size, number of typed SNPs, recombination rate) for HapMiner, SSA and MWU are 0.028 (0.0034), 0.045 (0.0047) and 0.022 (0.0027), respectively.

### The power of different methods

It is well known that a QTN can be easily detected by any (reasonable) method if it contributes a large fraction of the total variation in a phenotype, or if a very large sample size has been used. But in reality, for most complex traits, the contribution of a QTN to the phenotypic value is usually less than 10% of the total variance. On the other hand, the sample size in most studies is in the range of hundreds. In this study, we compare the power of the three methods under realistic assumptions that the QTN effect is not greater than 10% and that the sample size is not larger than 750 individuals.

#### The QTN effects and the sample sizes

**Figure 1:**
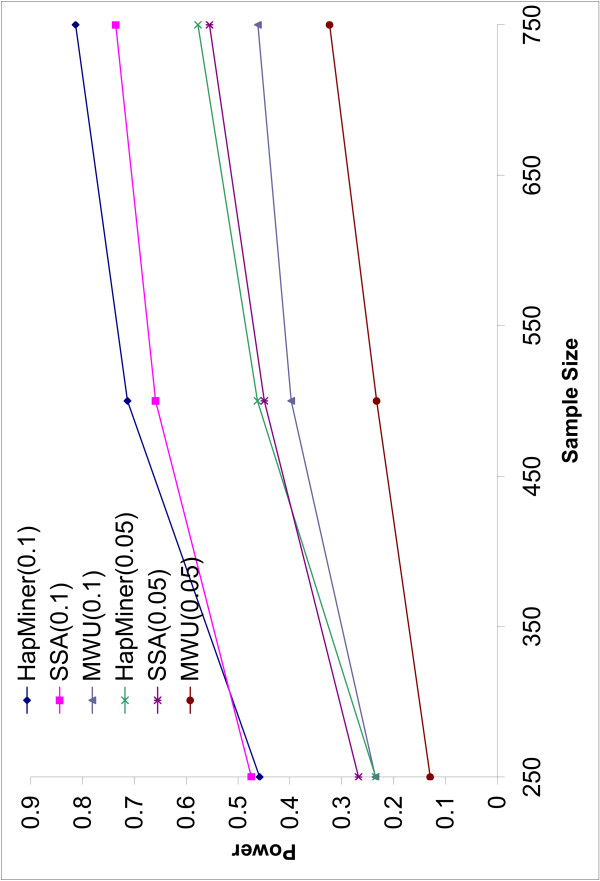
**Power vs Sample size**. The power of three methods as a function of sample sizes with two different QTN effects. Each point is the average power over all the recombination rates and different numbers of SNPs.

Figure 1 depicts the power of the three methods to detect significant associations when the QTN accounts for 10% and 5% of the total variation in phenotype, averaged over all other parameters such as recombination rates and the number of SNPs. As expected, the power of all three methods increases with the sample size and with increase of the QTN effect. These two factors have a bigger influence on power than the other factors. HapMiner and SSA achieve much higher power than the rank-based method (MWU) across all the parameters tested, demonstrating that much information is lost by only considering rank orders, and MWU should not be the choice in such an analysis. HapMiner is more powerful than SSA when the sample size is larger than 500. Otherwise, the two methods are comparable. The differences also depend on other parameters, such as recombination rates and marker densities, and will be discussed shortly. But in general, the power can be quite low (*<*50%) for all the methods if the sample size is smaller than 250 for both QTN effects. Even for a sample size of 750 individuals and with *π *= 10%, the average power of the three methods are only 81.3% (HapMiner), 71.3% (SSA) and 45.8% (MWU) (Figure 1). The values of the standard errors of power for both HapMiner and SSA are similar (around 0.05, not shown in Figure 1). This value is relatively large because the power of both methods can be quite different for different recombination rates or marker densities. Nevertheless, among the 36 different parameter combinations with the QTN effect of 10%, the power of HapMiner is higher than that of SSA in 33 cases with the largest difference of 13.5% (Table 1). In summary, for many quantitative traits with realistic effects, a larger sample size is necessary in real data analyses. In the following, we will mainly focus on the results based on 750 individuals with the QTN effect of 10%.

#### Recombination rates

**Figure 2:**
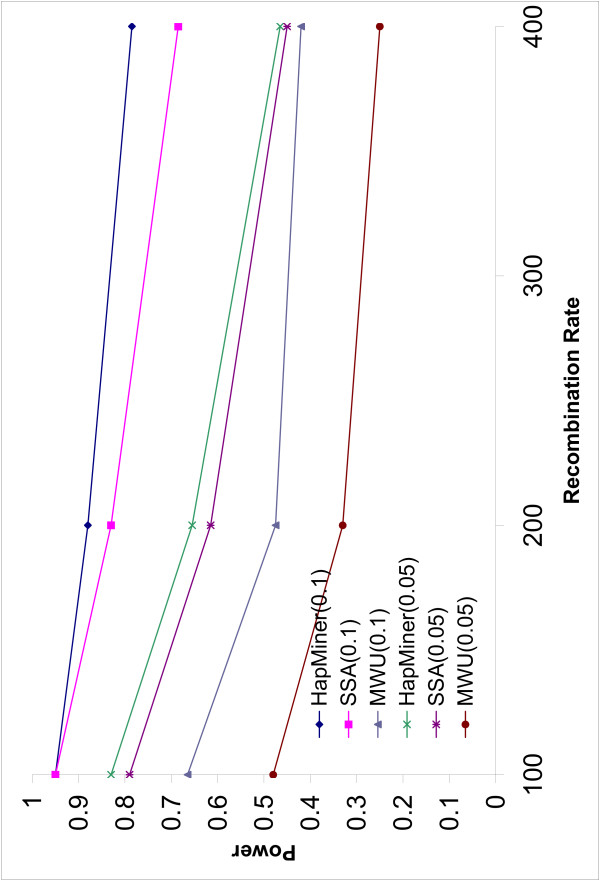
**Power vs Recombination rate (1)**. The power of three methods as a function of recombination rates when the sample size is 750. The average number of SNPs is 40.

**Figure 3:**
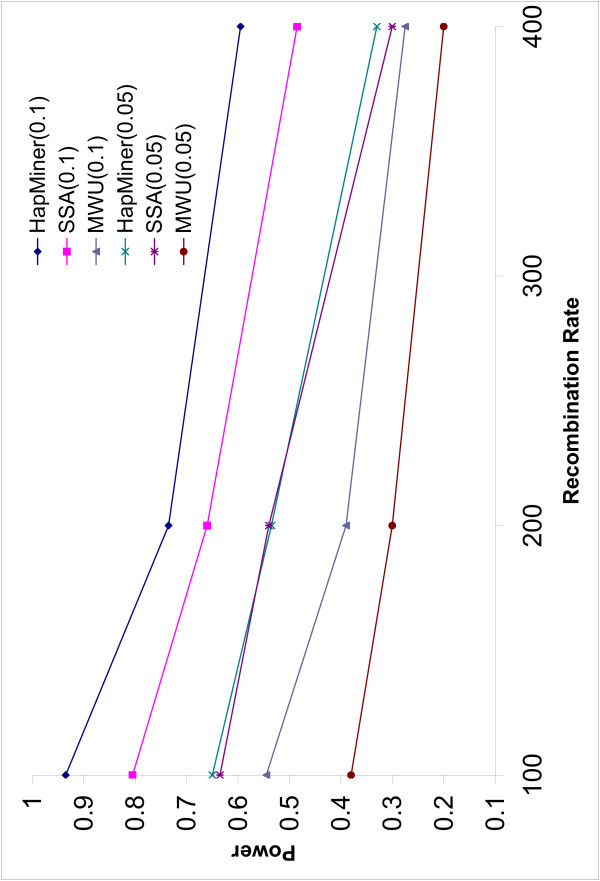
**Power vs Recombination rate (2)**. The power of three methods as a function of recombination rates when the sample size is 750. The average number of SNPs is 20.

The power of all three methods decreases with increase in the recombination rate (Figures 2, 3). This is not surprising because linkage disequilibrium breaks down more rapidly with larger recombination rates. HapMiner is more robust than the other two methods. The power of HapMiner has a smaller decrease than that of SSA if the density of the SNPs is high (Figure 2). HapMiner still consistently outperforms SSA across all the recombination rates for both marker densities when *π *= 0.1 (Figure 2, 3). The two methods achieve similar power when *π *= 0.05 for all recombination rates (Figure 2, 3). Recent human experimental data [6,13] indicate that the human genome can be partitioned into blocks of various lengths (tens to hundreds of kilobases) such that, within each block, there is no or little evidence of historical recombination events. In such regions with low recombination rates, it is not necessary to genotype every SNP. A small subset of tag SNPs can be used to reduce genotyping efforts without losing much information. Our results suggest that, in such a case, HapMiner has more advantages than SSA. For example, by using about half of the SNPs in the region with recombination rate of 100 (Figures 2, 3), the power of SSA dropped 15% (from 95.0% to 80.0%) while HapMiner only dropped 1.5% (from 95.0% to 93.5%).

#### Marker density

**Figure 4:**
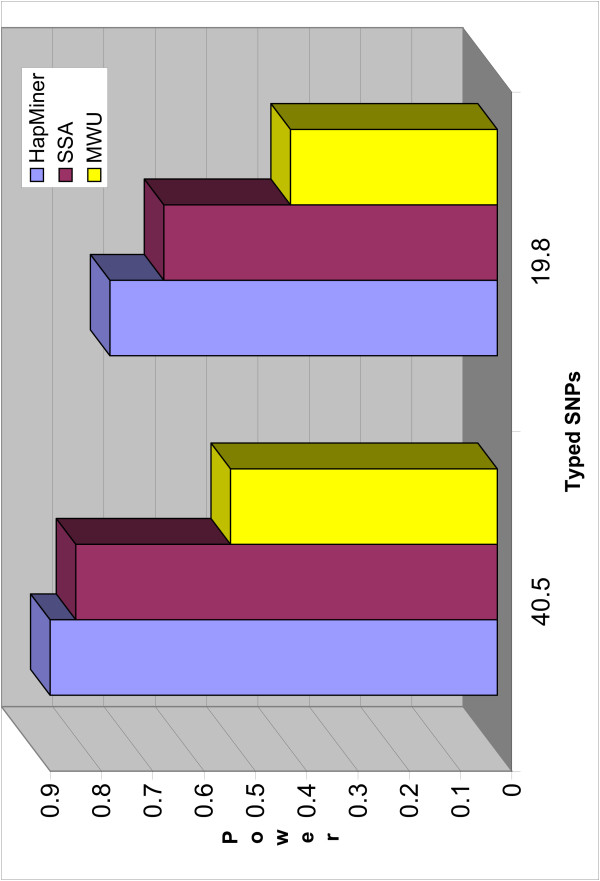
**Power vs Marker density**. The power of three methods as a function of the marker density, average over three recombination rates. The sample size is 750 and the QTN effect *π *= 0.1. The number of markers is the average number of SNPs (allele frequency ≥ 5%) over 200 replicates.

**Figure 5:**
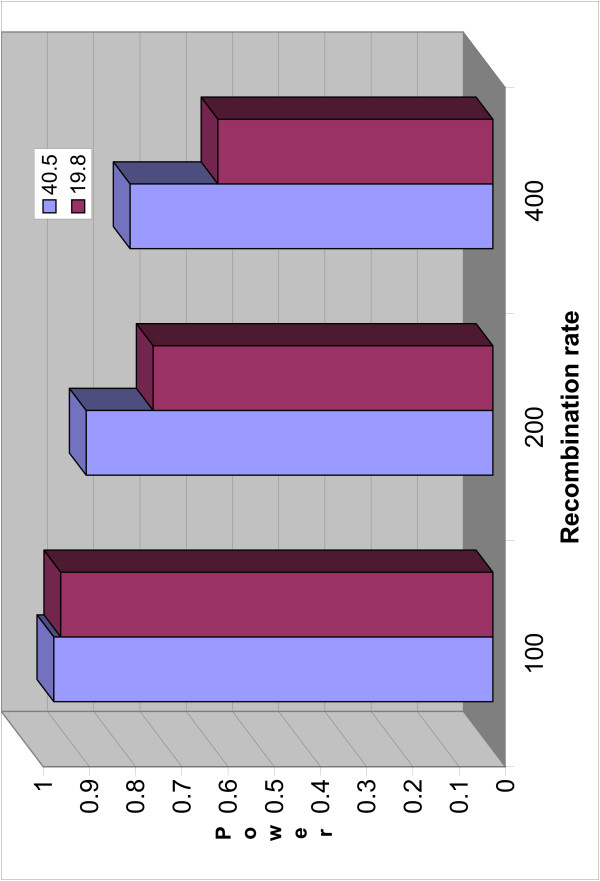
**Power of HapMiner with different recombination rates**. The power of HapMiner with different marker densities across different recombination rates.

**Figure 6:**
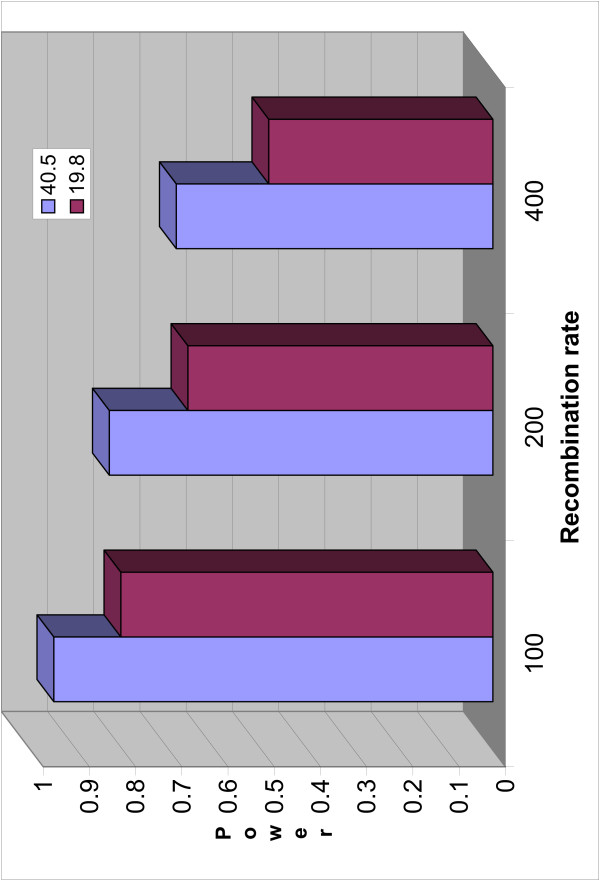
**Power of SSA with different recombination rates**. The power of SSA with different marker densities across different recombination rates.

The power of all three methods increases with increase in the number of typed SNPs. Figure 4 compares the power of the three methods when the region has different marker densities with the recombination rates being binned together. Figures 5 and 6 show the power of HapMiner and SSA with different marker densities for different recombination rates. For a small recombination rate (*u *= 100), the power of HapMiner only decreases little when the number of SNPs decreases by half, while SSA deteriorates much more. When the recombination rates are large (*i.e*., *u *= 200, 400), HapMiner gains more power than SSA on increasing the marker density. Overall, the increase of power for both methods is only small to modest when the number of SNPs is doubled. Therefore if resources are limited and the total number of genotypes to be typed (the number of individuals times the number of SNPs) is fixed in a given region for fine mapping, it is more desirable to have a large number of individuals with modest coverage. On the other hand, dense SNPs may provide more accurate information on location and this type of effect will be examined in the next subsection.

### Localization accuracy

We also investigate the prediction accuracy for each data set, when the identified association is significant, by taking the SNP with the highest score as the predicted QTN. Our simulation results show that the predictions are rather accurate for all three methods when associations are significant, especially when the sample size is large (*i.e*., 750). HapMiner performs consistently better than the other two methods. With high density markers, where the average number of SNPs in the analysis (with the minor allele frequency larger than 5%) is about 40.5 and the average marker interval distance is around 0.025, (*i.e*., 2.5% of the length of the region), the prediction errors of HapMiner are around 0.04 (within the range of 0.030 to 0.042). The accuracy increases with increase of the recombination rates. The predictions of the other two methods are also reasonably accurate (SSA: 0.040–0.053, MWU: 0.038–0.079). But no obvious trends are observed for these two methods when the recombination rate increases. When the marker interval distance is around 0.051 (*i.e*., around 19.8 markers in the region), the absolute values of prediction errors are larger than those with dense markers. But in terms of how many markers away the predicted positions are from the true QTNs, the results are comparable in these two cases. The prediction accuracy does not decrease substantially when the sample size decrease, or the QTN effect decrease, which illustrates that these two factors have most of their influence on power. Higher marker density improves the prediction accuracy in terms of absolute distances because the highest precision possible is half of the average marker interval distance. High recombination rates might give more accurate results, but with the risk of reduced power. The methods can be used as prediction tools because, under the simulated model, the association of SNPs with phenotype is mainly due to the linkage of the SNPs and the QTN. More investigations are needed for more complex population models.

### Results based on human data

The simulation based on coalescent theory might not capture the true property of LD in human populations owing to its assumptions of a simplified population structure and demographic history. We further test the three methods on empirical human data taken from [6]. The data consist of 129 trios in a region of 500 kb at 5q31 that is implicated as containing a genetic risk factor for Crohn disease. There is a total 103 SNPs with minor allele frequency *> *5%. The whole region shows a picture of discrete haplotype blocks with limited diversity within each block, suggesting great information redundancy. A substantially smaller subset of tag SNPs should be enough for association studies. We take the phased genotype data of all parents (in total 516 haplotypes) and mimic a two-stage study design for association mapping. At the first stage, only a small fraction of the total haplotypes is available to us, with all the SNPs. In this study, we randomly choose 150 haplotypes (around 30%) from the total of 516 haplotypes. The top 25 tag SNPs (around 25% of all the SNPs) are then selected using the online program Tagger [27], which has been demonstrated to be effective for SNP selection [7]. In the second stage, all the haplotypes with tag SNPs are then used in the power analysis. We randomly select a SNP from the tag SNPs as the QTN and the effect is set to be 0.1. A trait value for each haplotype is then generated according to the allele state of the QTN and the same phenotypic model as in the simulated data. The QTN is removed before further analyses. There are one hundred runs on each such data set and the power is defined as the proportion detecting significant associations. Table 2 summarizes the power of the three methods and the total numbers of genotypes screened under the two study designs. The two-stage design using tag SNPs can save more than half of the genotyping cost compared to the design with all SNPs. The power of HapMiner is almost the same for the two designs (from 88% to 87%). The power of SSA drops 9%, although it performs better than HapMiner when using all the SNPs. A possible explanation is that some SNPs are in almost complete LD with the QTN. In this case, taking the average over a haplotype segment like HapMiner does may actually deteriorate the power. MWU achieves better performance using tag SNPs than using all the SNPs. The reason for this is probably because the number of multiple tests for tag SNPs is much smaller than that for all SNPs. But its power is much lower than SSA and HapMiner. The results demonstrate that our haplotype-based approach has higher power when using tag SNPs than SSA and MWU, and should be used in studies with a two-stage design.

## Discussion

In this paper, we extend our previous haplotype-based association mapping method to quantitative traits. The algorithm has been implemented in our existing software called HapMiner. Extensive simulation results illustrate that HapMiner is more robust and achieves higher power than two other statistical approaches. The two methods (SSA and MWU) were chosen because of their popularity and their performance in previous studies [4]. We have not compared HapMiner with other haplotype-based approaches because of the lack of availability of existing programs for haplotype-based QTL association mapping using population samples.

In reality, most complex traits are the product of joint gene-environment action. The environmental factors may include, for example, smoking habit, drinking habit, times of exercise per week, special diet, among many others. Instead of using the *t*-based *Q*-score, we can easily incorporate the clustering algorithm into the framework of an ANCOVA analysis, thus taking into account environmental factors as well as gender, age, *etc*. as covariates into our haplotype-based association mapping model. More specifically, the marker information (all haplotypes) is taken as one independent variable, and all clusters (plus one more group formed by all random noise) are taken as the groups of that variable. ANCOVA (and a *F*-statistic) is used to test if the means of the groups are different enough not to have occurred by chance, with confirmation using a permutation test. If the result is significant, multiple comparison tests [24] can be employed to further test which groups/clusters are significantly different. By incorporating the clustering algorithm into ANCOVA framework, our approach has the potential to deal with locus or allelic heterogeneity. Because HapMiner can return multiple clusters at each marker, each of the clusters may represent a single ancestral mutation event. In such a case, ANCOVA (and an *F*-statistic) simultaneously tests if the mean values of different clusters and the group of random noise are the same. If the null hypothesis is rejected, multiple comparison tests [24] can be employed to further test which clusters are significantly different from the group of random noise. But a new permutation schedule is needed in this case and will be investigated in the future.

The method presented here assumes that haplotype information on each individual is available, which in general can be inferred based on genotype data using currently available programs (*e.g*. Haplotyper [23], Phase [26] for case-control data, or Genehunter [16], PedPhase [17] for case-parent data). The mapping accuracy directly depends on the quality of the haplotype inference. The trait value, which is the characteristic of an individual, is assigned to a pair of haplotypes, and the two haplotypes from one individual are assumed to be independent. All these factors may potentially compromise the effectiveness of the algorithm. An alternative to the use of inferred haplotypes is to calculate similarity/distance based on genotype vectors instead of haplotype segments. One way to extend the algorithm to genotype vectors is to consider the number of alleles that are *identical in state *(IIS) at each locus. The pair-wise similarity between genotype vectors can be defined by counting the number of alleles that are IIS and properly weighted. The clustering algorithm can then be applied to the genotype vector similarity matrix in the same way as we did before on the haplotype similarity matrix. But our preliminary results have shown that the method based on genotype vectors is not effective. On the other hand, it should also be noted that further (asymptotically) independent information is available in these vectors in the form of departure from Hardy-Weinberg equilibrium [25], and using this information may make the test more powerful. Another possible extension is that, for each individual, we consider multiple haplotype pairs that have high probability and are consistent with the genotypes. Further investigation will be needed on how to incorporate Hardy-Weinberg disequilibrium and/or the uncertainty during haplotype inference.

Marker selection is one key issue that will facilitate the process of identifying genetic contributions to complex traits. A number of methods have been proposed for identifying the subset with the minimum number of tag SNPs according to different metrics [6,13,30]. So the set of tag SNPs obtained by different methods may also be different. The discussion of the efficiency and power of different tag SNP selection approaches itself may require a separate paper. So in this paper, we have only considered different marker density in the simulated data and only one tag SNP selection method in the real data analysis. In the case where the haplotype block structure in a region is known in advance, it is also possible to take into consideration such prior knowledge. Instead of using the sliding window approach, one may perform tests block by block.

The *Q *score is based on the *t *statistic for comparison of means of different groups. One assumption of the *t *statistic is that it assumes equal variances in the two groups. This might not hold because similar haplotypes are more closely related and thus their trait values should be more similar to each other than haplotypes not forming any clusters. Therefore, it is expected that the variance of the trait values inside a cluster will be smaller. In this case, Welch's *T *statistic [24] for two independent samples with different variances can be adopted. Our tests have shown that the values of the two statistics are almost the same. However, since we are using a permutation test to obtain the significate level, and not the *t *distribution, the assumption of equal variances is not an issue for our algorithm.

It is well known that the spectrum of allele frequencies is also an important factor in determining the power of a method. The effect of allele frequency in this study can be easily seen from the model of generating phenotypic values in the Method section. With a low frequency, a QTN actually has a large allele effect; while with an intermediate frequency, it will have a smaller allele effect. The results presented in this paper comprise an average across different allele frequencies because each QTN is randomly chosen from all the SNPs with different frequencies. Finally, it should be noted that the permutation test for association is based on an exchangeability assumption. It is therefore important to have a sample from an ethnically homogeneous population or make allowance for the possibility of population stratification [26]. To what extent this might be an important issue will be studied in a future publication.

## Conclusion

In summary, HapMiner can be complementary to the current model-based statistical methods for QTL mapping and will serve as a useful tool for geneticists to explore their data. Our experimental results show that HapMiner is more robust and achieves higher power in most cases than two statistical approaches (SSA and MWU). The rank-based statistic (MWU) has much lower power than HapMiner and SSA, as shown in our study. In regions with low recombination rates or with blocks between recombination hot spots, two-stage association mapping using tag SNPs is an efficient study design to reduce genotyping cost without losing too much power. With the availability of HapMap data, such a design will gain much popularity in the near future. In such cases, HapMiner is preferable to SSA, as shown in this study, because haplotypes might capture moderate LD between tag SNPs in different blocks and haplotypes might represent some rare variants that will be missed by methods based on single markers using tag SNPs.

## Methods

The algorithm for a quantitative trait works as follows. The inputs to HapMiner are haplotypes, which can be inferred computationally based on information of family members or some population models for unrelated individuals. Both haplotypes of an individual take the same phenotypic value. For each marker position, a haplotype segment with certain length centered at the position is considered. Clusters are identified based on some similarity measure via a density-based clustering algorithm. For each cluster, a *Q*-score that is based on the *t*-statistic is calculated, representing the deviation of the phenotypic mean of the cluster from the phenotypic mean of all other samples. The *Q*-score can be used as an indicator of the degree of association between the cluster and the phenotype. The effectiveness of the method depends on the similarity measure of haplotype fragments, the clustering algorithm and the *Q*-score. We will describe each of these concepts shortly. The overall time complexity of our algorithm is *O*(*MN*^2^), where *M *is the total number of marker loci and *N *is the sample size, which is approximately in the hundreds in most real datasets. The algorithm is efficient for whole-genome screens, as shown in [18]. The current study focuses on QTL fine mapping.

### A haplotype sharing score

We have proposed a general haplotype similarity score in [18]. Briefly speaking, it is a combination of two similarity measures. One is the Hamming similarity and the other is the longest common substring. Since the similarity of two haplotype segments is defined with respect to a particular marker locus, we have introduced two weight functions based on the distance of a marker to the reference marker. The similarity measure is robust against recent marker mutations and genotyping/haplotyping errors, and it also picks up partial sharing from a common ancestral haplotype due to historical recombination events. We adopted the same similarity measure in the current study. In the following, we only illustrate the concept using an example. Detailed definition of the measure can be found in [18]. Suppose there are four haplotypes: *h*_1 _= (11212), *h*_2 _= (12222), *h*_3 _= (11221), and *h*_4 _= (21222), and we want to define similarities between *h*_1 _and *h*_2_, *h*_3 _and *h*_4_. If we only count the number of common alleles (Hamming similarity), both pairs have three common alleles, *i.e*., *s*(*h*_1_, *h*_2_) = *s*(*h*_3_, *h*_4_) = 3. But one might believe that *h*_3 _and *h*_4 _are more closely related because they share a longer segment which is more likely to be inherited from a recent common ancestor haplotype. If we define the similarity as the length of the longest common interval around the third locus in the middle, then *s*(*h*_1_, *h*_2_) = 0, *s*(*h*_3_, *h*_4_) = 2. But if there is a genotyping error or a point mutation from the ancestor haplotype at the second position of *h*_1_, the similarity of *h*_1 _and *h*_2 _will be underestimated. So we believe that, by combining these two measures, our similarity measure is more robust than either of them. Furthermore, we define the similarity of a pair of haplotypes with respect to each of the SNP positions. Weight functions can be naturally formulated based on the distance to the reference SNP. In our previous paper [18], we required the weight functions to be non-increasing functions of the genetic/physical distance but left it to users to choose their exact form. Another parameter that users must specify is the haplotype segment length. Although previous results [18] have shown HapMiner is robust to the selection of these parameters, it is still difficult to argue which values would be optimal. In this paper, we propose to use a pairwise linkage disequilibrium coefficient such as *D'*(*x*_0_, *x*_*k*_) between a locus *k *and the reference locus 0 as weights. HapMiner will automatically calculate the values of *D' *for each data set so users do not need to try different weight functions for different data sets. In addition, because the expected value of *D' *decreases with increase in marker distance, varying haplotype segment lengths might only have minimum influence on the final results in general, because SNPs with large distances are expected to contribute much less than nearby SNPs. In addition, in many studies, haplotype structures are actually defined based on a linkage disequilibrium measure such as *D'*. So the proposed new weights not only capture much information within a block, they can also incorporate some moderate linkage disequilibrium between blocks.

### A density-based clustering algorithm

Clustering is a powerful tool for mining massive data. The idea of assigning haplotypes to clusters for gene mapping is promising and has been explored by many researchers recently [10,18,19,22]. In the haplotype association mapping setup, we are interested in identifying haplotype clusters that are strongly associated with the quantitative trait under study. The goal is not to partition all the haplotypes into some clusters. Neither do we try to build a cladogram, because it is difficult to reconstruct the evolutionary relationship for all the haplotypes. Instead, we believe that for a quantitative trait locus with moderate heritability, mutant allele embedded haplotypes should have high similarity and their trait values may be significantly different from the values associated with the remaining haplotypes. A difficulty lies in the fact that, for many quantitative traits, there are many loci contributing to the trait but each only has small effects. Environmental influences further complicate the phenotype-genotype correlation and any explicit model would have difficulty in dealing with all the factors. We take the problem of finding strongly-trait-associated haplotype clusters as the problem of finding clusters from data with noisy background. We use the concept of "density-based clusters" and adopt an algorithm called DBSCAN [11] with minor modifications, which has been shown to be quite effective for disease gene mapping [18]. More details about the algorithm can be found in [11,18].

### Assessing the degree of association

Analogous to the *Z*-score used in disease gene mapping [18], we measure the degree of association with the trait under study using a *Q*-score, which is actually a *t*-statistic when we assume that the haplotypes in the cluster and the remaining haplotypes are sampled from two different populations. A large *Q*-score means strong association between the cluster (actually, the haplotypes within the cluster) and the trait. More specifically, let *m *denote the number of haplotypes in the cluster and let *n *denote the number of remaining haplotypes. Let μ^
 MathType@MTEF@5@5@+=feaafiart1ev1aaatCvAUfKttLearuWrP9MDH5MBPbIqV92AaeXatLxBI9gBaebbnrfifHhDYfgasaacH8akY=wiFfYdH8Gipec8Eeeu0xXdbba9frFj0=OqFfea0dXdd9vqai=hGuQ8kuc9pgc9s8qqaq=dirpe0xb9q8qiLsFr0=vr0=vr0dc8meaabaqaciaacaGaaeqabaqabeGadaaakeaaiiGacuWF8oqBgaqcaaaa@2E79@_*c *_and σ^c2
 MathType@MTEF@5@5@+=feaafiart1ev1aaatCvAUfKttLearuWrP9MDH5MBPbIqV92AaeXatLxBI9gBaebbnrfifHhDYfgasaacH8akY=wiFfYdH8Gipec8Eeeu0xXdbba9frFj0=OqFfea0dXdd9vqai=hGuQ8kuc9pgc9s8qqaq=dirpe0xb9q8qiLsFr0=vr0=vr0dc8meaabaqaciaacaGaaeqabaqabeGadaaakeaaiiGacuWFdpWCgaqcamaaDaaaleaacqWGJbWyaeaacqaIYaGmaaaaaa@30F4@ denote the sample mean and variance of the *m *haplotypes within the cluster and let μ^
 MathType@MTEF@5@5@+=feaafiart1ev1aaatCvAUfKttLearuWrP9MDH5MBPbIqV92AaeXatLxBI9gBaebbnrfifHhDYfgasaacH8akY=wiFfYdH8Gipec8Eeeu0xXdbba9frFj0=OqFfea0dXdd9vqai=hGuQ8kuc9pgc9s8qqaq=dirpe0xb9q8qiLsFr0=vr0=vr0dc8meaabaqaciaacaGaaeqabaqabeGadaaakeaaiiGacuWF8oqBgaqcaaaa@2E79@_*r *_and σ^r2
 MathType@MTEF@5@5@+=feaafiart1ev1aaatCvAUfKttLearuWrP9MDH5MBPbIqV92AaeXatLxBI9gBaebbnrfifHhDYfgasaacH8akY=wiFfYdH8Gipec8Eeeu0xXdbba9frFj0=OqFfea0dXdd9vqai=hGuQ8kuc9pgc9s8qqaq=dirpe0xb9q8qiLsFr0=vr0=vr0dc8meaabaqaciaacaGaaeqabaqabeGadaaakeaaiiGacuWFdpWCgaqcamaaDaaaleaacqWGYbGCaeaacqaIYaGmaaaaaa@3112@ denote the sample mean and variance of the *n *remaining haplotypes, respectively. The *Q*-score of the cluster is defined as:

Q=(μ^c−μ^r)m+n−2(σ^c2+σ^r2)(1/m+1/n).     (1)
 MathType@MTEF@5@5@+=feaafiart1ev1aaatCvAUfKttLearuWrP9MDH5MBPbIqV92AaeXatLxBI9gBaebbnrfifHhDYfgasaacH8akY=wiFfYdH8Gipec8Eeeu0xXdbba9frFj0=OqFfea0dXdd9vqai=hGuQ8kuc9pgc9s8qqaq=dirpe0xb9q8qiLsFr0=vr0=vr0dc8meaabaqaciaacaGaaeqabaqabeGadaaakeaacqWGrbqucqGH9aqpdaWcaaqaaiabcIcaOGGaciqb=X7aTzaajaWaaSbaaSqaaiabdogaJbqabaGccqGHsislcuWF8oqBgaqcamaaBaaaleaacqWGYbGCaeqaaOGaeiykaKYaaOaaaeaacqWGTbqBcqGHRaWkcqWGUbGBcqGHsislcqaIYaGmaSqabaaakeaadaGcaaqaaiabcIcaOiqb=n8aZzaajaWaa0baaSqaaiabdogaJbqaaiabikdaYaaakiabgUcaRiqb=n8aZzaajaWaa0baaSqaaGqaciab+jhaYbqaaiabbkdaYaaakiabcMcaPiabcIcaOiabigdaXiabc+caViabd2gaTjabgUcaRiabigdaXiabc+caViabd6gaUjabcMcaPaWcbeaaaaGccqGGUaGlcaWLjaGaaCzcamaabmaabaGaeGymaedacaGLOaGaayzkaaaaaa@56FB@

It is the scaled difference of population means from two samples, evaluated using sample means and variances, and follows approximately a *t*-distribution if we assume the trait values within the cluster and outside the cluster are independent, and both are normally distributed within groups with identical variances. This homoscedasticity assumption might not hold because similar haplotypes are more closely related, and thus their trait values should be more similar to each other, than haplotypes not forming any clusters. Therefore, it is expected that the variance of the trait values inside a cluster will be smaller. In this case, Welch's *T *test for two independent samples with different variances can be adopted. Notice that the proposed algorithm is non-parametric in nature and its significate level is obtained via a permutation test. The *t *distribution and the assumption of equal variances are not used directly in the algorithm.

### Permutation tests

The above algorithm takes a non-parametric framework to minimize the number of assumptions about the evolutionary history of the population, the genetic model of the complex trait, and the distribution of the quantitative value. Therefore, we generally do not assume the *Q*-score follows a *t *distribution. To assess the significance of the predicted gene position, a permutation test can be easily performed by shuffling the phenotypes among all the haplotypes to obtain an empirical *p*-value [5]. By randomly shuffling the phenotype values, it is expected that associations between haplotypes and the trait are broken. The association mapping analysis is performed on each shuffled data set and the values of the resulting statistics recorded. Then, the process is repeated for a sufficiently large number of times to mimic the permutation distribution of the original data. The proportion of the data sets whose statistic values are equal to or more extreme than the statistic produced by the original data set is regarded as the empirical *p*-value. The proposed method is computationally efficient so that permutation testing can actually be done even for a whole genome scan. This procedure also avoids the multiple testing problem that limits the power of any statistical test on a whole genome scale. The permutation test assumes an ethnically homogeneous population, or at least that the population can be divided into ethnically homogeneous strata within which the permutation can be done.

### Model for generating phenotypic data

For each replicate, a set of haploid individuals is generated from a fixed number of SNPs. A site with minor allele frequency larger than 5% is randomly chosen as the QTN. In generating the phenotypic values of each haploid individual, we adopt a distribution proposed in [20] using the following formula,

yi=(1−π)pi(1−pi)∗zi+π∗Qi,     (2)
 MathType@MTEF@5@5@+=feaafiart1ev1aaatCvAUfKttLearuWrP9MDH5MBPbIqV92AaeXatLxBI9gBaebbnrfifHhDYfgasaacH8akY=wiFfYdH8Gipec8Eeeu0xXdbba9frFj0=OqFfea0dXdd9vqai=hGuQ8kuc9pgc9s8qqaq=dirpe0xb9q8qiLsFr0=vr0=vr0dc8meaabaqaciaacaGaaeqabaqabeGadaaakeaaieGacqWF5bqEdaWgaaWcbaGae8xAaKgabeaakiabg2da9maakaaabaGaeiikaGIaeGymaeJaeyOeI0ccciGae4hWdaNaeiykaKIaemiCaa3aaSbaaSqaaiabdMgaPbqabaGccqGGOaakcqaIXaqmcqGHsislcqWGWbaCdaWgaaWcbaGaemyAaKgabeaakiabcMcaPaWcbeaakiabgEHiQiabdQha6naaBaaaleaacqWGPbqAaeqaaOGaey4kaSYaaOaaaeaacqGFapaCaSqabaGccqGHxiIkcqWGrbqudaWgaaWcbaGaemyAaKgabeaakiabcYcaSiaaxMaacaWLjaWaaeWaaeaacqaIYaGmaiaawIcacaGLPaaaaaa@4EC0@

where *π *is the proportion of variation attributable to the QTN, *p*_*i *_is the allele frequency, *z*_*i *_follows the standard normal distribution, and *Q*_*i *_is the number of mutant alleles (0 or 1). The distribution basically assumes that the heritability due to this particular QTN is *π *and the proportion of all other variation due to the environment or other genes (under an additive model) is 1 - *π*. It can be seen that the real allele effect of the QTN depends on both the allele frequency and the heritability *π*. In our simulation, the results are for the average across different allele frequencies because each QTN is randomly selected for each replicate.

## Authors' contributions

JL initiated, designed, and carried out the research and drafted the manuscript. YZ participated in the discussion and gave inputs to improve the manuscript. RCE provided suggestions on the statistical analysis and discussion.

## Figures and Tables

**Table 1:** Power of HapMiner and SSA (*π *= 0.1). The power of HapMiner and SSA across all the parameter combinations when the QTN effect is 0.1.

Sample size	# of SNPs	40.5			19.8		
	
	Rec. rate	100	200	400	100	200	400
750	HapMiner	95.0	88.0	78.5	93.5	73.5	59.5
	SSA	95.0	83.0	68.5	80.0	66.0	48.5
500	HapMiner	90.5	80.0	59.0	82.0	69.5	47.0
	SSA	87.0	78.5	57.5	76.0	58.5	38.0
250	HapMiner	62.0	53.5	43.5	58.0	36.5	21.5
	SSA	62.0	54.5	40.0	59.5	36.5	32.0

**Table 2:** Comparison of the three methods on the real data set. The power of the three methods when using all SNPs or using tag SNPs only. The numbers in parentheses are the total numbers of genotypes screened for the two designs.

Design (# of Genotypes)	HapMiner	SSA	MWU
All SNPs (26574)	88%	93%	33%
Tag SNPs (12300)	87%	84%	45%
